# Emission Characteristics of Heat Recirculating Porous Burners With High Temperature Energy Extraction

**DOI:** 10.3389/fchem.2020.00067

**Published:** 2020-02-11

**Authors:** Abhisek Banerjee, Alexei Saveliev

**Affiliations:** Department of Mechanical and Aerospace Engineering, North Carolina State University, Raleigh, NC, United States

**Keywords:** porous combustion, combustion emissions, NO_x_ formation, combustion kinetics, pollutant kinetics

## Abstract

Emission characteristics of heat recirculating porous burners with high temperature heat extraction are studied numerically. Two types of burners are considered: counterflow porous burner (CFB) and reciprocal counterflow porous burner (RCFB). The combustion of methane-air mixtures flowing through the porous media is modeled by solving steady state governing equations to obtain the flame temperature and species profiles. Formation of CO, NO, NO_2_, and NO_x_ is studied in CFB and RCFB in a range of equivalence ratios from 0.3 to 1.0 and heat extraction temperatures from 300 to 1,300 K. The contribution of various NO formation mechanisms is comparatively analyzed and related to the NO generation predicted by a detailed chemistry mechanism. The effect of high temperature heat extraction on the formation of CO and NO_x_ is analyzed. Numerical predictions indicate a constant monotonic decrease of NO_x_ concentration with increasing temperature of energy extraction. The formation of CO is observed to follow the similar trend. For heat extraction at 1,300 K, simulations predicted 3.6 ppm of NO_x_ and 3.9 ppm of CO for CFB and 4.1 ppm of NO_x_ and 3.5 ppm of CO for RCFB when these burners are operated at an equivalence ratio of 0.7.

## Introduction

Oxides of nitrogen (NO and NO_2_), also termed as NO_x_, are well-known to be detrimental to the environment. Starting from ozone-depletion, photochemical smog to acid rain, these chemical compounds are responsible for many adversities to the environment and human life. NO_x_ are generated from automobiles and industries involving thermal power generation and boilers. Nitrogen as a main air component is inevitably present in all the combustion systems. This makes combustion process a major contributor to the total concentration of atmospheric NO_x_. In last few decades, many researchers have been working to study the mechanism of NO_x_ generation in flames (Marteney, 1970; Iverach et al., 1973; Bowman, 1975; Miller and Bowman, 1989).

The formation of NO in combustion commonly follows three main routes: thermal (Zeldovich) mechanism, the prompt (Fenimore) mechanism and N_2_O-intermediate mechanism. In addition, some researchers reported NO formation through NNH pathway (Bozzelli and Dean, 1995; Harrington et al., 1996; Hayhurst and Hutchinson, 1998; Klippenstein et al., 2011). Zeldovich (1946) described the NO formation through reactions *N*_2_ + *O* → *NO* + *O*, *N*_2_ + *O* → *NO* + *N*. He reported these reactions to be slower than other reactions taking place during combustion. The rate of NO formation is controlled by the second reaction, owing to the high activation energy of 314 kJ/mol. As a result, Zeldovich mechanism displays a strong dependence on temperature. Miller and Bowman (1989) confirmed that this mechanism is insignificant below 1,800 K. Fenimore (1971) presented a prompt mechanism closely linked with the combustion chemistry of hydrocarbons. He proposed a series of reactions leading to the fast formation of NO. NO formation through this mechanism takes place in the reaction zone. He concluded that the concentration of CH radicals is a significant factor affecting the total NO formation. Prompt mechanism is responsible for NO_x_ formation at low temperatures (Dupont and Williams, 1998; Steele et al., 1998). As the name suggests, N_2_O-intermediate NO formation proceeds through a set of reactions involving N_2_O (Miller and Bowman, 1989).

Despite the formation mechanisms of NO_x_ are fairly well-known, intrinsic control of its minimization is still a subject of extensive research. In this context, Takeno et al. (1981) and Kotani and Takeno (1982) devised the concept of inserting solid porous medium in the reaction zone of a premixed flame for achieving heat recirculation. They reported higher burning rates compared to that of free flames and observed lower emissions of CO and NO_x_. Since then, many researchers (Khanna et al., 1994; Ellzey and Goel, 1995; Kennedy et al., 2002; Bingue et al., 2007; Bubnovich et al., 2007) have studied porous medium combustion using various designs of the burners to observe low emissions of CO and NO_x_. Recently, Banerjee et al. (2019) studied a two-stage combustion system (combination of filtration and non-premixed combustion) and reported low NO_x_ emissions. The unique characteristics of filtration combustion, such as strong interfacial heat transfer between solid and gas phase and enhanced gas phase dispersion of reactants and products (Kennedy et al., 2000), create a foundation for stable combustion over a wide range of reactant velocities, and fuel-air ratios. These attributes of filtration combustion have led to its potential applications in several domains like coating and paint drying, metal heat treatment, hydrogen and syngas synthesis, electricity generation (Marbach and Agrawal, 2005; Toledo et al., 2011, 2012; Bubnovich et al., 2016; Banerjee and Saveliev, 2018, 2019). Depending on the type of application, researchers have used various design of burners filled with porous medium like counterflow porous burner (CFB) (Belmont and Ellzey, 2014; Banerjee and Saveliev, 2018), reciprocal flow burner (RFB) (Contarin et al., 2003), and a combination of counterflow and reciprocal flow porous burners called as reciprocal counterflow porous burner (RCFB).

One of the novel applications of heat regenerating porous burners is related to portable power generation systems based on thermoelectric and thermionic generators and Stirling engines. The burners are incorporated in these systems to supply high temperature heat to electricity generators. High energy efficiency and low emission levels are the major requirements for the combustion devices used. Heat recirculating porous burners are the perfect candidates. The high energy efficiency in these burners is achieved by the internal heat regeneration. They are also known to have ultralow emission characteristics because of the extended low temperature combustion zones. Previous studies reported the effects of equivalence ratio and firing rate on the emission characteristics of the heat recirculating porous burners (Kennedy et al., 2002; Afsharvahid et al., 2008). However, the effect of the high temperature heat extraction on NO_x_ and CO formation was not considered.

This article studies NO_x_ and CO formation in a CFB and RCFB when heat is extracted from them at high temperatures. Various NO formation mechanisms are comparatively analyzed to understand the contribution of individual pathways to the total NO_x_ generation. The effect of the heat extraction temperature on NO_x_ and CO formation is considered to address feasibility of these burners for applications in portable power generators and other combustion systems.

## Numerical Model

### Model Geometry

**Figure 1:**
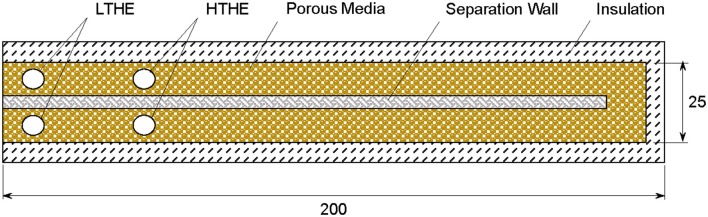
Model geometry and schematic of the porous burner.

Two burners, namely CFB and RCFB, are studied for high temperature heat extraction. To simplify a comparative analysis, the numerical model considers the same physical geometry for the porous burner operating in CFB and RCFB modes. The two-dimensional burner (Figure 1) has an active length of 200 mm and height of 25 mm. The separation wall splits the burner in two channels. The wall transfers the heat between two channels providing heat recirculation between hot products and cold reactants. The wall is made of alumina. The porous medium is formed by a packed bed of solid spheres resulting in a porosity of ~0.4. The uniform porosity distribution is assumed by the model. The burner contains four tubular heat exchangers placed in the locations shown by circles in Figure 1. The heat exchangers are treated as walls maintained at a fixed temperature. The low temperature heat exchangers (LTHEs) operate at 300 K and positioned near the inlet and outlet of the burner. LTHS are always active and help to restrict flame inside the burner. The high temperature heat exchangers (HTHEs) operate at extraction temperatures varying from 300 to 1,300 K. HTHEs are activated based on the burner operation mode as described below. The placement of HTHEs is selected based on numerical optimization to achieve maximum energy extraction efficiency (Banerjee and Saveliev, 2018; Banerjee, 2019).

### CFB and RCFB Operation

**Figure 2:**
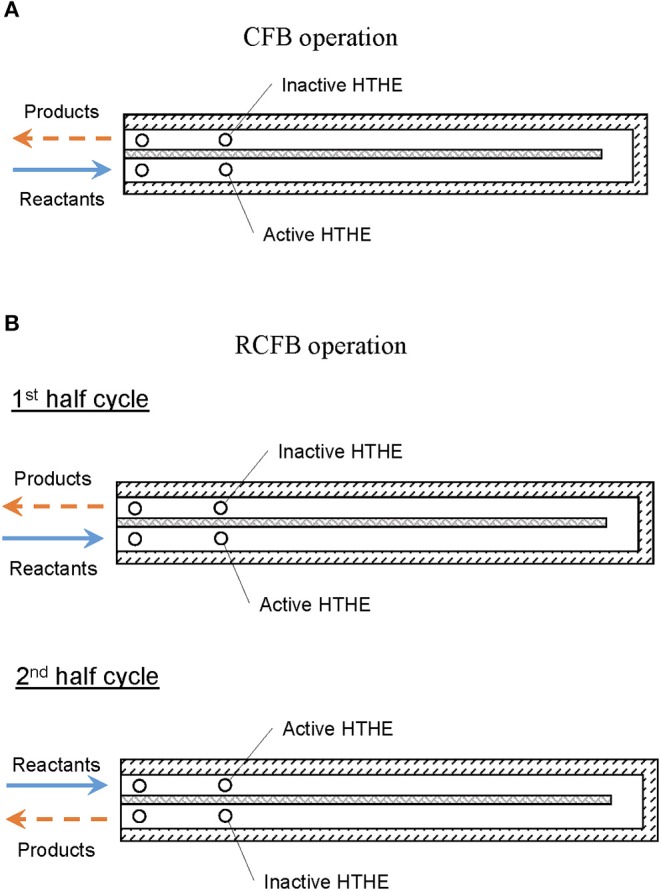
Schematic representation of **(A)** CFB and **(B)** RCFB operation modes. Arrows indicate direction of flow.

CFB is named to reflect the direction of fluid flow in the burner. During its passage through the burner, the fluid passes through a counter flow path (Figure 2A). The fuel/air mixture enters the inlet channel and gets preheated until it reaches the flame zone. The hot products leave the burner through the exhaust channel. The counterflow arrangement of fluid flow, results in heat regeneration between hot products and cold reactants.

Periodic switching of the direction of reactant flow is the basic principle of operation for a reciprocal flow burner. This flow reversal through the burner filled with solid porous medium, results in heat regeneration (Contarin et al., 2003). Using the concept of periodic flow reversal in CFB, a new type of burner named as RCFB was proposed by Banerjee (2019). In a RCFB, the direction of fluid flow is altered periodically to regenerate heat more efficiently than in a CFB. During flow reversal, the outlet of the burner is switched to inlet. This switching results in more efficient preheating of the reactants. The operation of RCFB is shown schematically in Figure 2B.

### Computational Model

The model used to simulate combustion in CRB and RCFB is based on the following assumptions: (i) the pressure drop in the burners is negligible, (ii) the gas and solid phases are in thermal equilibrium, (iii) radiation heat transfer in the gas phase is negligible compared to that in the solid phase, (iv) porous medium is chemically inert.

The numerical simulation of CFB is based on steady state solution of governing equations for a fully developed flow through porous medium. The details of the continuity, momentum, energy, and species equations is provided by Banerjee and Saveliev (2018). For this study, the numerical model accounts for conduction, convection, and radiation heat transfer.

RCFB burner works on the principle of heat regeneration by periodic flow reversal in a counterflow porous burner. The numerical model to study the unsteady behavior of a system having periodic flow reversal is very computationally expensive. Hence, to simulate quasi-steady behavior of RCFB, a time-averaged approach (Yao and Saveliev, 2018) is adopted. The time-averaged computational model of an RCFB system considers two CFBs having opposite directions of the gas flow and placed in thermal contact with each other. This model able to mimic the quasi-steady state behavior of the RCFB system. Detailed description of the computational model RCFB is available elsewhere (Banerjee, 2019).

A set of boundary conditions is imposed on the numerical model for simulating combustion and high temperature heat extraction. The inlet for the burner is treated as velocity inlet. The superficial velocity of the reactant mixture kept constant at 0.36 m/s for this study. The outlet of the burner is set as pressure outlet. The external burner walls are considered adiabatic. The heat extraction from the burner through heat exchangers is calculated based on temperature difference between adjacent cells. The value thus obtained, is integrated over the complete domain of the heat exchanger.

### Chemical Mechanism

Chemical mechanism plays a significant role in simulating combustion process in a burner. Be it evolution of temperature inside the burner or concentration of chemical species, chemical mechanism contributes significantly in a numerical simulation. Single-step chemical mechanisms have the advantage of predicting the combustion process in relatively lesser time than detailed mechanisms. However, they lack accuracy in many aspects such as species concentrations. In order to study the chemistry of NO_x_ production, a detailed chemical mechanism is required for accurate prediction of temperature profile and concentration of various chemical species formed as a result of combustion. Hence, for this study one of well-known detailed chemical mechanisms GRI 3.0 (Smith et al., 2009) is used for simulating combustion. This mechanism comprises of 53 chemical species and 325 reactions.

### Solution Procedure

The numerical simulations in this study are performed using Fluent 14.5. The governing equations are solved with steady-state approximation using a pressure-based solver. This solver uses an iterative approach to achieve convergence through a continuous loop. Absolute velocity formulation is used to predict combustion numerically. The numerical simulation is performed in laminar flow regime. Pressure and velocity are coupled using SIMPLE scheme. The energy, momentum and species equations are discretized using second order upwind scheme.

A grid independence study performed on numerical models of CFB and RCFB shows strong variation of maximum flame temperature when the number of grids was in a range from 200 thousand to 400 thousand. However, as the number of grids is increased beyond 600 thousand, the variations decreased largely. For CFB the stable grid independent temperature was obtained beyond 600 thousand, however, for RCFB the same is reached for grids with more than 800 thousand cells. Hence, the numerical simulations for CFB and RCFB were conducted with 860 thousand and 925 thousand cells, respectively.

## Results and Discussion

**Figure 3:**
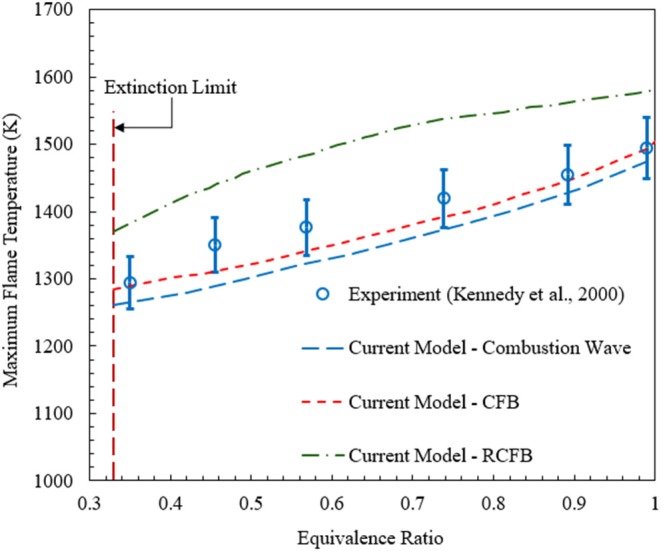
Maximum flame temperatures predicted by the numerical model for CFB, RCFB, and freely propagating combustion waves. Experimental data for freely propagating combustion waves in a tubular reactor (Kennedy et al., 2000) are plotted for comparison. The superficial velocity is equal to 0.36 m/s.

Flame temperature is one of the most important characteristics of the porous medium combustion. Figure 3 shows the maximum flame temperature predicted by the current model for CFB, RCFB, and a freely propagating combustion wave in the tubular burner configuration studied by Kennedy et al. (2000). The numerical predictions for the maximum flame temperature in Kennedy's burner agree well with the experimental data reported by Kennedy et al. (2000). In porous combustion with heat recovery, the maximum flame temperature shows only weak dependence on the energy content of the mixture. The flame zone is free to position itself in the porous medium to achieve optimal heat recirculation. The porous medium conducts and radiates the heat from the flame zone to the relatively cooler zones inside the burner. This process of heat transfer restricts a significant rise in maximum flame temperature with increase of the equivalence ratio. In contrast to homogeneous flames, the maximum combustion temperature is mainly defined by the kinetics of combustion and heat transfer characteristics. Figure 3 also shows the maximum flame temperatures for CFB and RCFB. These temperatures are very close to the temperatures for the freely propagating wave and demonstrate only moderate increase with rise in equivalence ratio. For a specific equivalence ratio, the maximum flame temperatures are predicted for RCFB and almost the same for the CFB and the freely propagating combustion wave. This difference is mainly attributed to the effect of flow reversal in the RCFB.

**Figure 4:**
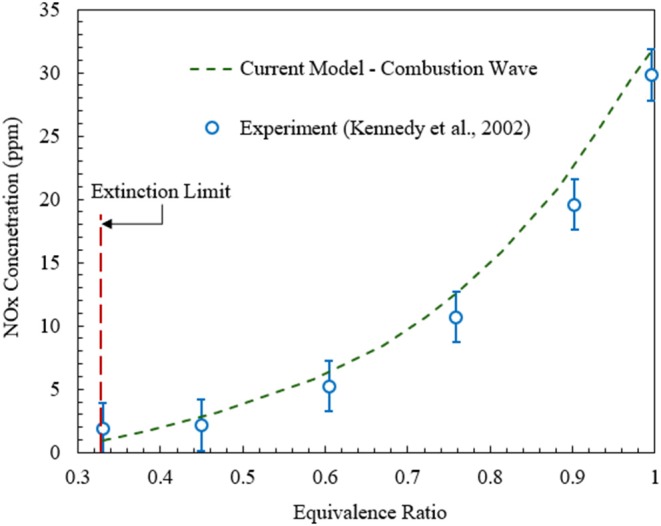
NO_x_ concentrations predicted by the numerical model for freely propagating combustion waves in comparison with the experimental data (Kennedy et al., 2002).

It is also important to validate model predictions for NO_x_ against published experimental data. The comparison of the numerical predictions for the freely propagating combustion wave model and experimental data (Kennedy et al., 2002) is shown in Figure 4. Numerical simulations show that for ultralean combustion NO_x_ formation is insensitive to the change in equivalence ratio. However, for ϕ > 0.4 NO_x_ concentration increases rapidly with the rise in equivalence ratio. Overall, as confirmed by Figures 3, 4, the numerical predictions of the model used in this study agree well with the experimental results in terms of the maximum flame temperature and NO_x_ concentration.

**Figure 5:**
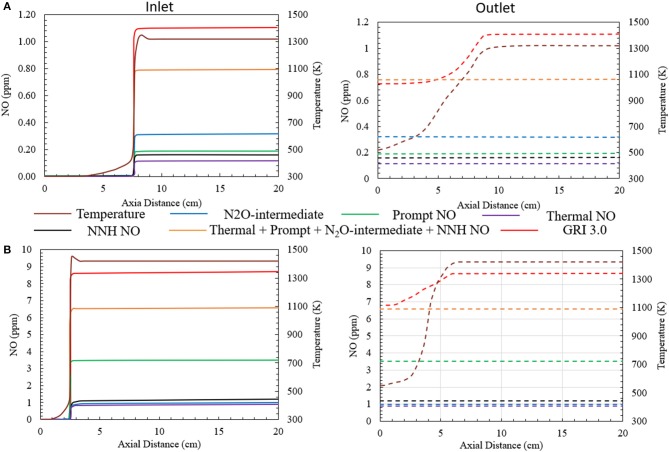
NO formation via various major NO generating mechanisms for **(A)** ϕ = 0.4 (ultralean mixture) and **(B)** ϕ = 0.7 (lean mixture). Superficial velocity of reactants is maintained at 0.36 m/s. The HTHEs are inactive.

Major NO formation routes have been studied for heat recirculating porous burner using numerical predictions. The study is performed for the CFB operating at ϕ = 0.4 (ultralean) and ϕ = 0.7 (lean). The HTHEs are not activated. In Figure 5, the NO profiles are shown along the axial direction of the inlet and outlet channels. The left-hand side represents the inlet channel and the right-hand side shows the outlet channel. The contribution of various NO formation mechanisms toward the total NO emitted by the burner is shown. The NO concentrations generated by individual mechanisms, the total NO concentration (thermal + prompt + N_2_O + NNH), and the NO concentration predicted by GRI 3.0 are reported.

For the ultralean condition (ϕ = 0.4), the NO concentration predicted by the numerical model is slightly less than 1 ppm (0.73 ppm) at the burner outlet by GRI 3.0. The NO formation through thermal, prompt, N_2_O-intermediate and NNH mechanisms is shown in Figure 5A. The N_2_O-intermediate mechanism is dominant. The prompt and NNH mechanisms jointly contribute comparable amount of NO. However, the contribution of thermal NO mechanism is negligible compared to other pathways studied here. The direct summation of all the NO generation pathways leads to the NO concentration of 0.77 ppm (Figure 5A) well below the predictions of GRI 3.0 for the high temperature region of the burner. However, the NO concentrations predicted by the chemical mechanism combining the individual NO generating mechanisms differ from GRI 3.0 by a very insignificant margin of ~0.05 ppm. Concurrently, simulating this ultra-lean porous combustion with GRI 3.0 mechanism exhibits a decrease in the concentration of NO near the exit of the burner where the temperature drops. This is mainly because a part of the NO formed inside the burner transforms to NO_2_ before exiting the burner. As a result, GRI 3.0 predicts NO formation of 0.73 ppm at the exit of the burner.

Figure 5B shows similar plot for the combustion of a lean methane/air mixture at ϕ = 0.7. In this case, the numerical simulation predicts an increase in the concentration of NO formed inside the burner. The prompt NO formation mechanism is observed to be the major contributor to the total NO formation. The NO concentration formed through the prompt mechanism is close to 3.5 ppm. This is mainly because the high concentration of hydrocarbons in the reactant mixture increases the concentration of C, CH, and CH_2_ radicals that govern NO formation through the prompt mechanism. Whereas, the other mechanisms like thermal, N_2_O-intermediate and NNH mechanism produce approximately 1 ppm NO individually. The summation of all the NO generating mechanisms leads to a total NO concentration of 6.5 ppm. GRI 3.0 predicts 6.65 ppm of NO at the burner outlet. Corresponding to the previous case, GRI 3.0 mechanism shows a decrease in the NO concentration near the outlet of the burner, owing to the conversion of NO to NO_2_. Likewise, the deviation between the NO formation predicted by the combination of all the NO generating mechanism and that by GRI 3.0 in observed to be insignificant.

GRI 3.0 mechanism predicts a decrease in NO concentration near the burner outlet. The NO generation occurs mainly in the flame zone, with a negligibly small fraction generated in the post flame region. The temperature near the burner outlet decreases. This decrease in the temperature leads to NO being converted to NO_2_. Bowman (1975) reported the primary conversion reaction as *NO* + *HO*_2_ ↔*NO*_2_ + *OH*. The difference in the concentration of total NO (thermal + prompt + N_2_O + NNH) and GRI 3.0 is related to the NO conversion near the outlet. The numerical concentrations of NO reported at the manuscript correspond to the NO concentration predicted by GRI 3.0 at the outlet of the burner.

**Figure 6:**
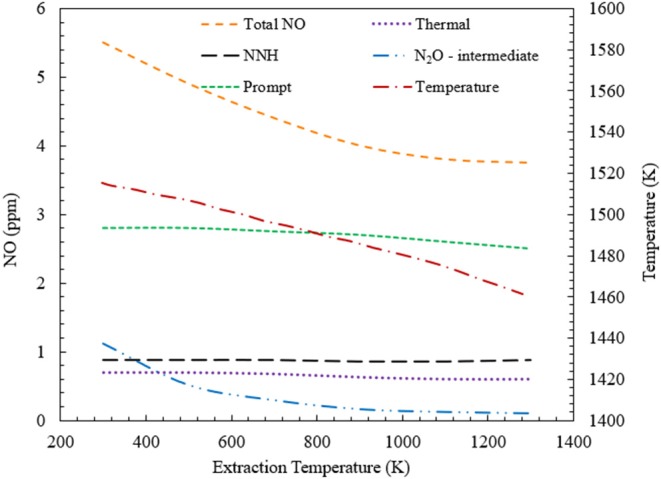
Contribution of various NO formation mechanisms toward the total NO formation inside a CFB. This variation is studied for CFB when the energy extraction temperature is varied from 300 to 1,300 K at ϕ = 0.7. Numerical simulation predicts a decrease in total NO concentration, with increase in the heat extraction temperature.

Figure 6 shows the variation of NO generation through four major pathways, when heat is extracted from the CFB. For ϕ = 0.7, the heat extraction temperature is varied from 300 to 1,300 K for the CFB. Numerical simulation predicts a decrease in the total NO formation with increase in heat extraction temperature. This decrease is mainly driven by the reduction of NO formed through N_2_O-intermediate mechanism. However, there is a little decrease in the NO formation through the prompt mechanism. This is explained in detail in the following section of the manuscript. For CFB operating at ϕ = 0.7, the NO concentration is predicted to drop from 5.5 ppm to nearly 4 ppm.

**Figure 7:**
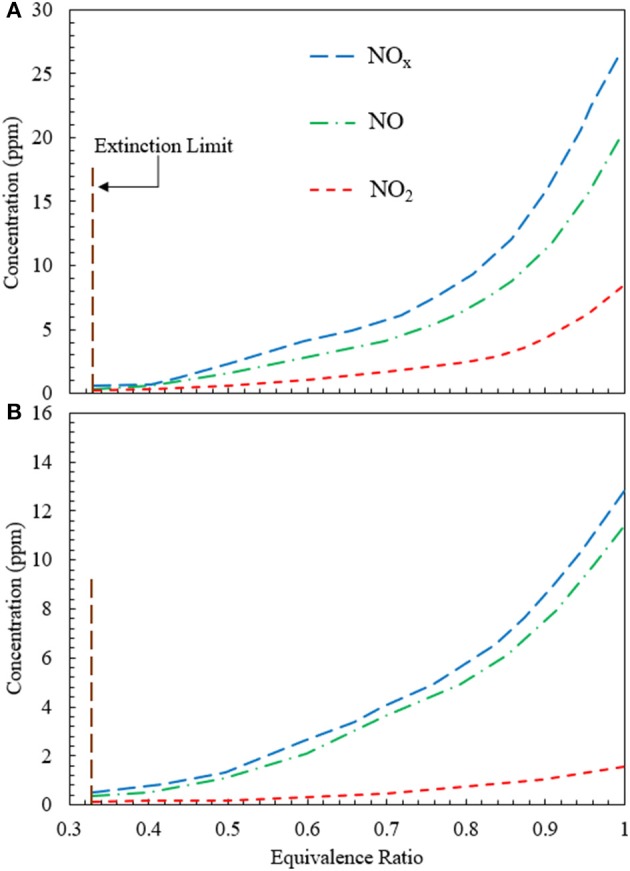
Variation of NO, NO_2_, and NO_x_ concentration generated in the CFB when energy is extracted at **(A)** 500 K and **(B)** 1,100 K. The concentrations of NO, NO_2_, and NO_x_ rise when the equivalence ratio is increased from the extinction limit (ϕ ≈ 0.33) to stoichiometry (ϕ = 1). The superficial velocity of reactant mixture is 0.36 m/s.

The variation of NO_x_ concentration as a function of equivalence ratio is studied for CFB when heat is extracted at 500 and 1,100 K (Figure 7). For the extraction at 500 K, NO concentration increases monotonically from 0.4 ppm at ϕ = 0.3 to 18.2 ppm at ϕ = 1.0. The increase in NO remains insignificant until ϕ = 0.4. However, for 0.4 < ϕ < 1.0, NO concentration demonstrates a steady increase. The variation of NO_2_ appears to be similar to the NO profile. However, the concentration of NO_2_ is lesser than that of NO for the entire range of equivalence ratios studied. Numerical simulation predicts that NO_2_ concentration varies from 0.1 ppm at ϕ = 0.33 to 8 ppm at ϕ = 1.0, as shown in Figure 7A. The variation of NO_x_ concentration remains similar to that of NO and NO_2_. For CFB with heat extraction at 500 K, the NO_x_ concentration varies from 0.5 to 26.2 ppm for 0.33 < ϕ < 1.0. Figure 7B shows the plot for NO_x_ concentration when heat is extracted at 1,100 K. Similar to the previous case of heat extraction at 500 K, the variations of NO, NO_2_, and NO_x_ show similar trend. However, for extraction at 1,100 K, the concentrations of NO, NO_2_, and NO_x_ is lower than that at 500 K. Numerical simulations show that for the heat extraction at 1,100 K, the concentration of NO_x_ increases from 0.4 to 11.65 ppm at the range of equivalence ratios from 0.33 to 1.0.

**Figure 8:**
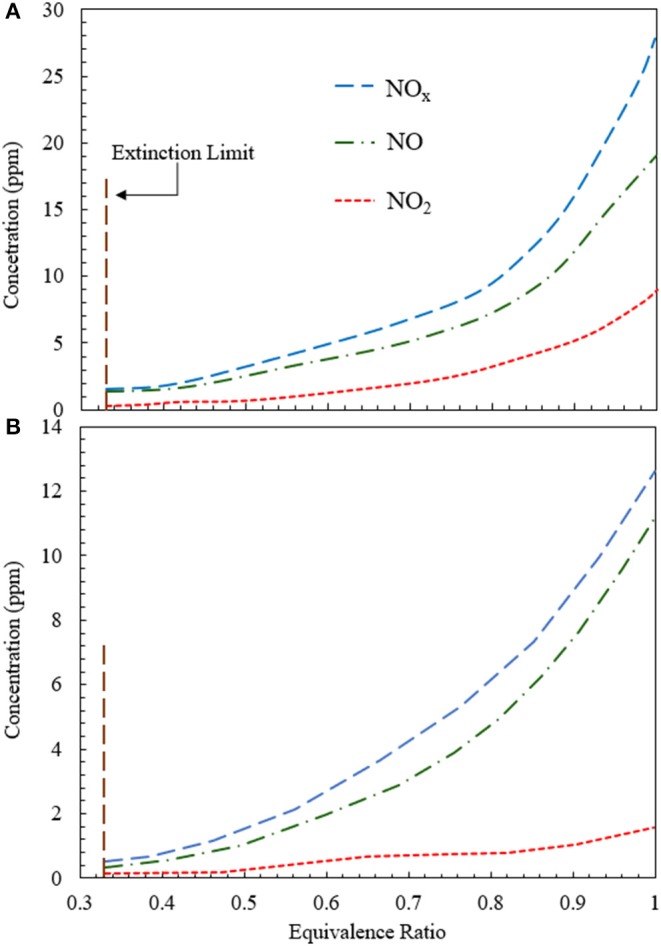
Variation of NO, NO_s_, and NO_x_ concentration for RCFB as a function of equivalence ratio when heat is extracted at **(A)** 500 K and **(B)** 1,100 K. The concentrations of NO, NO_2_, and NO_x_ increase with rise in equivalence ratio from the extinction limit (ϕ ≈ 0.33) to stoichiometry (ϕ = 1). The superficial velocity of reactant mixture is kept at 0.36 m/s.

Figure 8 illustrates the variation of NO_x_ concentration for heat extraction at 500 and 1,100 K using an RCFB. Figure 8A shows that NO_2_ concentration does not change appreciably for 0.33 < ϕ < 0.5. After ϕ = 0.5, NO_2_ concentration increases monotonically. Numerical simulation predicts NO_2_ values in the range from 0.2 to 7.8 ppm for 0.33 < ϕ < 1.0. The NO concentration varies from 0.33 to 20.2 ppm and that of NO_x_ increases from 0.5 to 27.9 ppm for 0.33 < ϕ < 1.0 (Figure 8A). The concentrations of NO, NO_2_, and NO_x_ are lower for heat extraction at 1,100 K than that at 500 K. Numerical model shows that NO_2_ concentration varies between 0.1 and 1.7 ppm, NO concentration remains within the range from 0.3 to 11.1 ppm and NO_x_ concentration increases from 0.4 to 12.8 ppm when the equivalence ratio is increased from 0.33 to 1.0 (Figure 8B).

**Figure 9:**
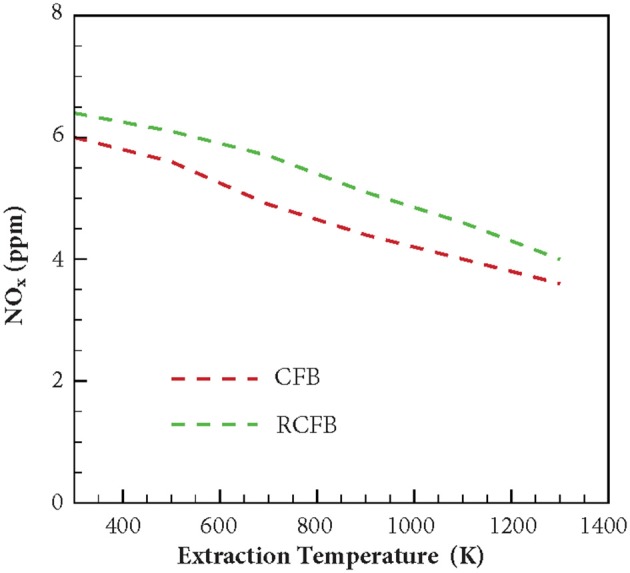
Variation of NO_x_ concentration for CFB and RCFB as a function of energy extraction temperature. NO_x_ concentration decreases for CFB and RCFB with increase in energy extraction temperature. The burners are operated at ϕ = 0.7 with a superficial velocity of 0.36 m/s.

Figure 9 shows the variation of NO_x_ generated in CFB and RCFB as a function of the heat extraction temperature. The study is conducted for CFB and RCFB operating at ϕ = 0.7. Numerical predictions show that NO_x_ concentration decreases from 6 to 3.6 ppm for CFB when the heat extraction temperature is increased from 300 to 1,300 K. Similarly, for RCFB the amount of NO_x_ generated decreases from 6.4 to 4.1 ppm for the same range of heat extraction temperatures. It is interesting to note that the amount of NO_x_ generated for RCFB is more than that of CFB for the entire range of heat extraction temperatures.

**Figure 10:**
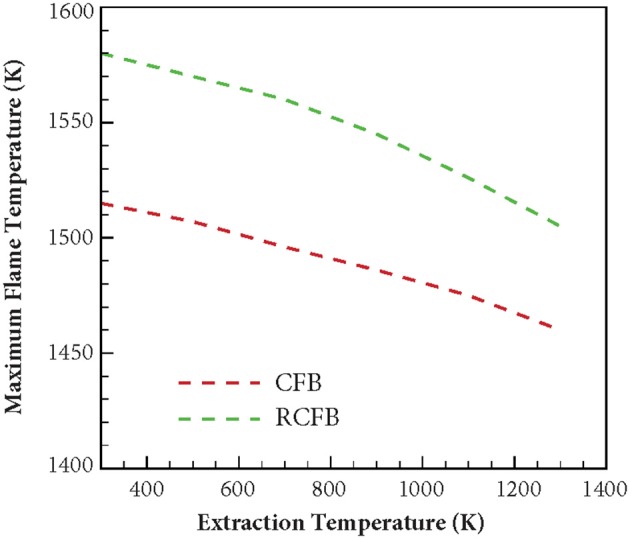
Variation of maximum flame temperature for CFB and RCFB when heat is extracted at high temperature. Maximum flame temperature decreases with increase in heat extraction temperature in a way similar to decrease in NO_x_ concentration. The burners are operated at ϕ = 0.7 with a superficial velocity of 0.36 m/s.

The variation of maximum flame temperature of CFB and RCFB is shown in Figure 10. For both CFB and RCFB the maximum flame temperature decreases with increase in the heat extraction temperature. In case of CFB this decrease in maximum flame temperature is between 1,515 and 1,460 K when the heat extraction temperature rises from 300 to 1,300 K. However, for RCFB this decrease in maximum flame temperature is from 1,580 to 1,505 K. Thus, it can be inferred that for the entire range of heat extraction temperatures studied here, the maximum flame temperature for RCFB is higher than that of CFB. This results in higher NO formation through thermal mechanism in RCFB. Since the equivalence ratio of the fuel mixture entering the burner is the same for CFB and RCFB, the decrease in NO_x_ formation in CFB is the result of lower NO formation through thermal mechanism in CFB than that in RCFB. A comparison of Figures 9, 10 shows that the variations of NO_x_ concentration for CFB and RCFB with heat extraction temperature follow a trend similar to the one followed by maximum flame temperature vs. heat extraction temperature for the burners.

**Figure 11:**
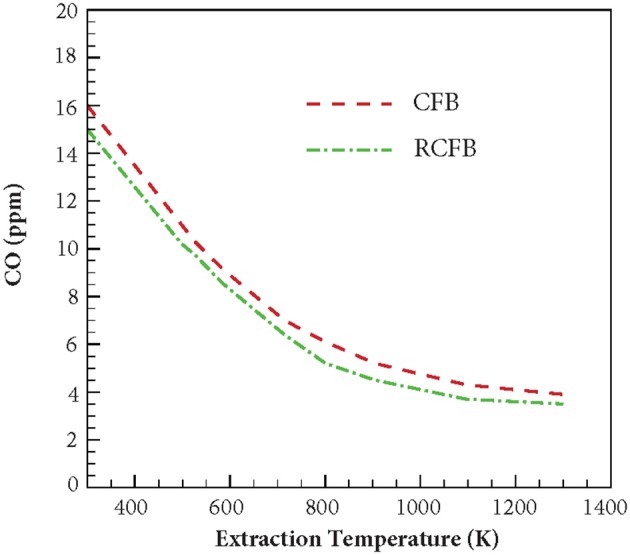
Variation of CO concentration generated in CFB and RCFB as a function of energy extraction temperature. The burners are operated at ϕ = 0.7 with a superficial velocity of 0.36 m/s.

Similar to NO_x_, variation of CO concentration is studied for CFB and RCFB when the heat extraction temperature is varied between 300 and 1,300 K (Figure 11). The burners are operated with fuel/air mixture at ϕ = 0.7. For CFB, increasing the heat extraction temperature from 300 to 1,300 K lowers the CO formation from 16 to 3.9 ppm. Whereas, energy extraction in RCFB within this temperature range results in CO concentration to drop from 15 to 3.5 ppm. Similar to the trend of NO_x_ formation, CO concentration reduces with increase in the heat extraction temperature.

**Figure 12:**
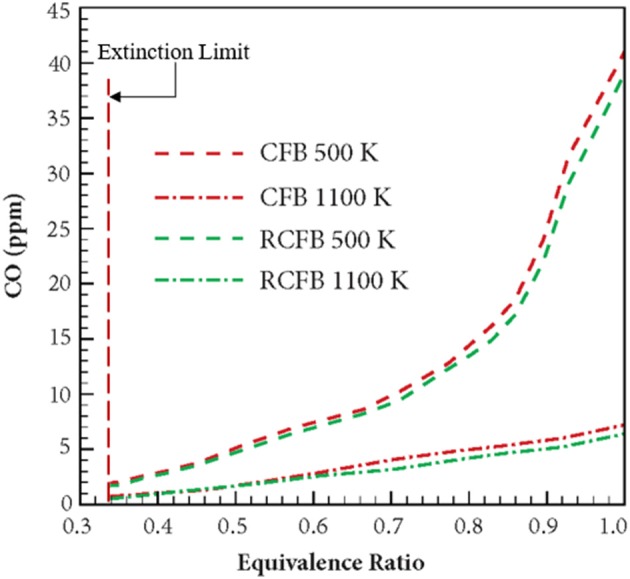
Variation of CO concentration in CFB and RCFB with equivalence ratio. Numerical study is performed for heat extraction at two temperatures: 500 and 1,100 K. Concentration of CO is observed to increase with an equivalence ratio of the methane/air mixture from the extinction limit (ϕ ≈ 0.33) to stoichiometry (ϕ = 1). The burners operate with a superficial velocity of 0.36 m/s.

Figure 12 shows the variation of CO concentration for CFB and RCFB as a function of equivalence ratio when energy is extracted at 500 and 1,100 K. For energy extraction at 500 K, the CO concentration increases from 1.4 to 41 ppm when the equivalence ratio increases from 0.33 to 1.0. This trend remains unchanged for RCFB as the CO concentration is observed to increase from 1.2 to 39 ppm. This increase of CO concentration can be attributed to the increase in hydrocarbon concentration in the fuel mixture with increase in equivalence ratio. Similarly, for energy extraction at 1,100 K, the CO concentration increases from 0.6 to 7.2 ppm for CFB and 0.4–6.4 ppm for RCFB when equivalence ratio is increased from 0.33 to 1.0. Similar to the behavior of NO_x_ concentration, CO concentration is observed to decrease when the heat extraction temperature increases. The trend remains valid for the range of equivalence ratio studied in this work.

## Conclusions

This article presents the results from numerical investigation of the emission characteristics of CFB and RCFB when energy is extracted from these burners at high temperature. Various NO generation routes are analyzed to establish the relative contribution of these mechanisms in the total NO concentration emitted from the burner. It is found that amongst the four major NO producing mechanisms namely thermal mechanism, prompt mechanism, N_2_O-intermediate mechanism and NNH pathway, the N_2_O-intermediate mechanism is the major contributor for NO formation when ultralean mixtures are burned. However, for the combustion of lean mixtures prompt mechanism gains dominance over other routes. This is mainly because increase in the hydrocarbon concentration raises the concentration of C, CH, and CH_2_ radicals, which are major chemical species responsible for NO generation through prompt mechanism. The simulation results for the CFB and RCFB show that the NO_x_ generated by RCFB is more than that of CFB by approximately 1 ppm in all the cases studied here. This is mainly because of the higher maximum flame temperatures for RCFB than CFB leading to higher NO_x_ formation through thermal mechanism. It has been observed that with increase of energy extraction temperatures, NO_x_ concentration decreases for both heat recirculating porous burners studied here. This is attributed to the decrease in the maximum flame temperatures of CFB and RCFB with an increase in heat extraction temperature. This decrease in temperature diminishes the contribution of N_2_O-intermediate mechanism and prompt mechanism and ultimately lowers the total NO_x_ generated. Numerical results predicted that the maximum flame temperatures decreases in a way similar to the NO_x_ concentration. Similar variation is observed for CO concentration. The results indicate a decrease in CO concentration with increase in heat extraction temperature for both CFB and RCFB. Numerical simulations predict an increase in CO formation with rise in the equivalence ratio for both heat recirculating porous burners studied. This increase is due to rise in the hydrocarbon concentration at high equivalence ratios.

## Data Availability Statement

The datasets generated for this study are available on request to the corresponding author.

## Author Contributions

All authors listed have made a substantial, direct and intellectual contribution to the work, and approved it for publication.

### Conflict of Interest

The authors declare that the research was conducted in the absence of any commercial or financial relationships that could be construed as a potential conflict of interest.
